# Editorial

**DOI:** 10.4102/sajp.v73i1.438

**Published:** 2017-11-30

**Authors:** Aimée V. Stewart

**Affiliations:** 1 Department of Physiotherapy, University of the Witwatersrand, South Africa

I had the privilege of being appointed as the editor-in-chief of the *South African Journal of Physiotherapy* in March this year. I follow a long line of editors, each of whom has worked hard to improve the standing of the journal. I hope that with the profession’s help I can continue the advancement of our journal.

One of the most noteworthy changes to the journal occurred under the editorship of Prof. Josè Franz and that was the appointment of AOSIS (Pty) Ltd as the publisher. This has meant that the journal is now an online journal and has the support of a professional, experienced and enthusiastic organisation. This makes the future of the journal more secure and enables more progress to be made in improving the standard of the journal.

Our profession has grown in leaps and bounds over the years in South Africa (and with much hard work!).

We have gone from a diploma qualification at many of the training institutions to a 4-year bachelor’s degree and increasing numbers of physiotherapists now have higher degrees. These are both master’s and doctoral degrees. This means that there is an increasingly strong cohort of researchers and clinician researchers who are developing a local evidence base for our practice. These are the people we need to tap into to encourage them to consider this journal for their work. Quite rightly they want to publish in the best possible journals so it is incumbent on the editorial team to make this journal one of the best available.

So where do we go from here? My aim over my term as the editor is to increase the quality and the visibility of the journal and ensure that it becomes a journal of choice for potential authors – obviously not the only journal that they consider for publication. That means that the journal must continue to be locally relevant whilst increasing its continental and international standing. Academics, in particular, need to publish in a variety of journals to ensure that they and their work reach as wide an audience as possible. So to make our journal one that they would consider as a first option means that much work needs to be done to improve both the quality and visibility of the journal.

Steps were recently taken by Prof. Franz and the AOSIS (Pty) Ltd team to register the journal on PubMed, and the process is nearing its completion, with what we hope will be a positive outcome. This means that if we are registered, our articles will be listed on PubMed making our authors’ work internationally available. The next step is to get the journal listed on Medline and other international listings. This will require much work. The standard and number of submissions need to improve, and we need to get a wider, more international authorship and readership.

To enable the above listings, we have already started on a number of endeavours to comply with the requirements to be listed. Firstly, our editorial board has been increased and now includes local, continental and international experts. The members of the board will help to advertise the journal amongst their colleagues, to increase both the readership and submission number. Secondly, we intend to solicit articles from a variety of experts that will be of value to our readership and at the same time increase the quality of the journal. Whilst doing this I am very conscious of the fact that we need to remain a friendly, supportive journal for young researchers in order that they may publish their articles with us. I hope that I, together with the editorial board, can provide this support. The satisfaction of publishing for the first time is one that we must support, as it is hoped that we will be facilitating these researchers to continue to publish.

We also need to provide education and support for our reviewers so that we build up a strong body of competent reviewers. We have recently re-invited the whole list of reviewers to be on the reviewers’ list to ensure that those on the list are there because they want to support the journal. The minimum qualification to be a reviewer is to have a master’s degree and two publications. If readers feel that they would like to become reviewers and they have the above qualifications, they must please contact us so that they can be added to the list. At some point, we will have to consider running a course in reviewing and editing, I believe.

We have also started on a programme to increase the readership of the journal. We would really like our profession to read and be aware of what is being done research-wise in our own environment so as to include local studies in their evidence-based practice. This programme includes a Twitter feed as well as a variety of advertisements at appropriate congresses. For example, we had a wonderful poster on the South African Society of Physiotherapy stand at the World Confederation of Physical Therapy in July this year. Other ideas are being considered and will be put in place in due course.

Our journal has been in existence for over 40 years. This is an amazing statement of the dedicated professionals who have guided the profession over so many years. So our publishers have embarked on a programme to add all previous issues to the electronic database thus ensuring that this wonderful heritage will be there for future generations of physiotherapists. It also provides readers with the opportunity to see how the profession has grown and changed over the years.

So there is much work to be done! I am sure that with the support of the profession, we can build on our solid foundation that has been developed over the years by many dedicated editors and their teams, so that we become a journal that is locally relevant but also has international standing, readership and submissions of articles.

